# Patterns and associated factors of COVID-19 knowledge, attitude, and practice among general population and health care workers: A systematic review

**DOI:** 10.1177/2050312120970721

**Published:** 2020-11-11

**Authors:** Firomsa Bekele, Tadesse Sheleme, Ginenus Fekadu, Kumera Bekele

**Affiliations:** 1 Department of Pharmacy, College of Health Science, Mettu University, Mettu, Ethiopia; 2 School of Pharmacy, Institute of Health Science, Wollega University, Nekemte, Ethiopia; 3 Department of Nursing, College of Health Science, Selale University, Fiche, Ethiopia

**Keywords:** Coronavirus disease 2019, knowledge, attitude, practice, associated factors

## Abstract

**Introduction:**

Coronavirus disease 2019 is a pandemic disease, requiring persons around the world to take immediate action to reduce the risk of infection. This study was aimed to summarize the patterns and determinants of coronavirus disease 2019 knowledge, attitude, and practice among general populations and health workers.

**Methods:**

A cross-sectional study from PubMed, HINARI, and Scopus were searched from March 16 to July 30, 2020. The review was done in line with the Preferred Reporting Items for Systematic Reviews and Meta-analyses–2009.

**Result:**

We found 56 articles upon the initial search. Finally, 21 studies were filtered to be studied in this systematic review. Overall, the majority of the articles that were previously published had good knowledge about coronavirus disease 2019 that lies in the ranges from 40% to 99.5%. A good attitude lies in the ranges from 70% to 97.1%. Among impact of coronavirus disease 2019 on mental health, only anxiety was reported that ranges from 24.6% to 96.3%. We found the variable practice towards combating coronavirus disease 2019. Several factors were associated with poor knowledge, attitudes, and practice skills regarding the pandemic of coronavirus disease 2019 such as level of education, occupation, income, gender, age, residence, work experience, religion, having media, marital status, and race.

**Conclusion:**

The majority of the articles that were previously published had found good knowledge and attitude about coronavirus disease 2019 and variable reports for practice to combat the disease. Most of them were severely worried about the disease. Therefore, the mental effect of the coronavirus disease 2019 should be studied at large, and every country should implement the strategy to combat the disease to increase the level of practice.

## Introduction

Coronavirus disease 2019 (COVID-19) is a respiratory infection caused due to a novel coronavirus (SARS-COV-2) and was first observed in Wuhan, China, and the disease has a fatality rate of 2.3%.^1^ The clinical presentation includes fever, dry cough, fatigue, myalgia, and shortness of breath.^2–4^

Currently, the disease became a pandemic in the majority of the countries, requiring persons around the world to attend to updated information about the disease and apply the recommendations to tackle the risk of infection.^5,6^ On the last January 2020, the World Health Organization (WHO) declared that the disease is to be a public health emergency.^7^

The disease is widely transmitted via fluid droplets, individuals touching their mouth, nose, or eye mucosa with their hands, coughing and sneezing, and touching a material that the virus on it.^8,9^ There was the greatest risk of COVID-19 transmission to health care providers. Therefore, it is paramount to protect health care providers to maintain the care of the patients and to minimize the spread of the disease to other clients.^3^

Despite most of the COVID-19 is self-limiting, some patients have presented with different complications including organ damage, shock, lung parenchymal infections, acute respiratory distress syndrome (ARDS), venous thromboembolism, and pulmonary embolism^3,7,10,11^

Currently, there is no approved treatment for the coronavirus despite multiple researches has been conducted in many clinical trials.^12^ Therefore, prevention is the mainstay of therapy to combat the disease.^13^

Despite multiple trials has been done to avoid the disease, the success or failure of these efforts largely relies on the behavior of the clients.^14,15^ People’s observance of the management strategy is indispensable for combating the transmission of COVID-19, which is affected by their knowledge, attitudes, and practices (KAP) toward COVID-19.^16^

KAP studies give vital information to decide the best intervention programs to change misunderstanding about the disease.^17^ Besides this, it can help program planner to evaluate their policy toward improving people’s awareness of the disease.^18^

Knowledge of disease may influence the behavior of health care providers.^19^ Similarly, public knowledge is indispensable to avoid the disease. Therefore, determining the behavior of the population and health care providers can help to dig out their perception and practice toward the COVID-19.^13^ Therefore, this review tried to summarize the KAP of COVID-19 among the general population and health workers across the globe.

## Methods

### Data sources and searching procedure

This review aims is to summarize the published articles on the KAP and associated factors of COVID-19. This study was conducted according to Preferred Reporting Items for Systematic Reviews and Meta-analyses (PRISMA)-2009.^20^ The articles were searched by three reviewers (F.B., T.S.H., and G.F.) and the fourth author (K.B.) was consulted for disagreements of the significance of the studies to be included in the review.

We searched studies in Medline via PubMed, HINARI, and Scopus were included in the final analysis according to the inclusion criteria mentioned. The period included was from the March 16 to July 30, 2020. We checked the references of retrieved studies for additional studies manually. Endnote x5 was used to remove exact duplicates and to manage our library.

The search terms for each database were as follows: (Knowledge AND attitude AND practice AND COVID-19 OR Associated factors) OR (knowledge AND attitude AND practice AND SARS-CoV-2) OR (knowledge AND attitude AND practice AND MERS-VOV) OR (knowledge AND COVID-19) OR (attitude AND COVID-19) OR (Practice AND COVID-19).

### Eligibility criteria

Articles on COVID-19 of human studies published in the English language, which contain relevant outcomes, were included. Adult studies which met the preceding criteria are eligible. Initially, we obtained 56 articles using a systematic search on the database. After duplicates (9 articles) was removed, 16 were removed due to their title were not consistent (either narrow or broad) to our study and the abstract was incomplete and full texts were not available. The remaining 10 articles were excluded due to unclear outcomes of interest, preprints, and letters (short communication). Finally, 21 articles that met the inclusion criteria were included in the synthesis.

### Data abstraction

The authors filtered the articles from eligible studies onto a data abstraction sheet. We extracted information on the name of first author and year of publication, country, study designs, number of participants, average age (years), gender, occupation, educational level, outcome endpoints, and their associated factors.

### Methodology quality assessment

The National Institutes of Health (NIH) Quality Assessment tool was used to determine the quality of the studies. Accordingly, each question was answered with “yes,” “no” or “cannot determine” and” not applicable” and “not reported.”^21^

## Results

### Search results

**Figure 1.**
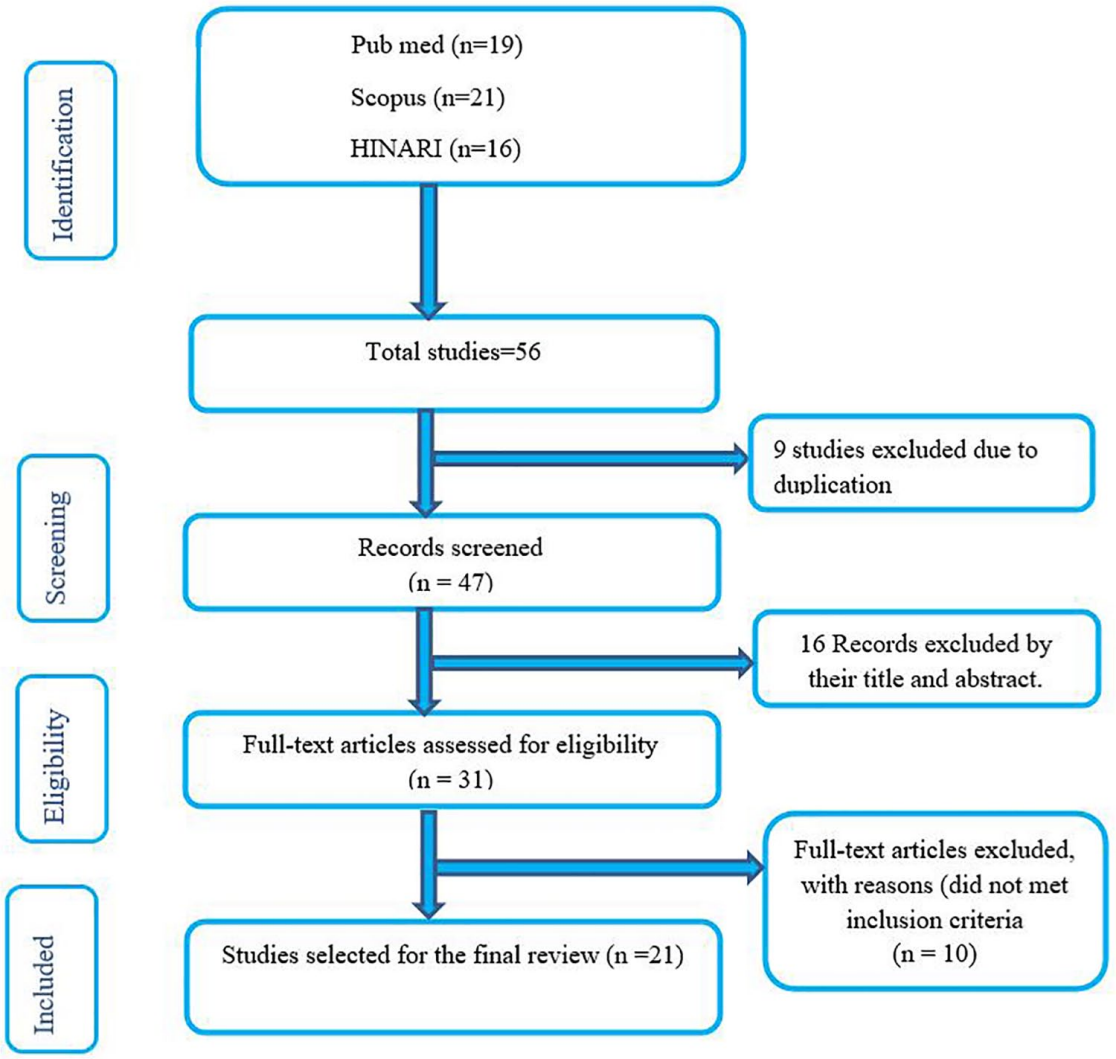
Flow chart of the systematic research and study selection process.

Initially, 56 publications were obtained from three databases (PubMed, HINARI, and Scopus). After the removal of nine duplicates, the remained articles were 47. We excluded 16 articles by reviewing their titles and abstracts. As a result, only 31 articles were subject to a full-text review. Finally, 21 articles that fulfilled eligible criteria were included in the review (Figure 1).

### Characteristics of studies included in this review

All articles were cross-sectional studies. Articles included in this study were undergone in 14 countries China, United States, India, Turkey, United Kingdom, Iran, Malaysia, Vietnam, Jordan, Pakistan, Nigeria, Philippines, Qatar, and Saudi Arabia. The review was conducted from March 16 to July 30, 2020. The study conducted in China indicated that regarding their occupations, the majority of the participants were psychiatrists accounts 141 (45.34%) and nurses were 70 (54.66%).^22^ The study participants of Turkey showed that specialist accounts 175 (50.6%), resident were 117 (33.8%), professor were 19 (5.5%), associate professor was 14 (.0%), and the assistant professor was 21 (6.1%).^8^ Similarly, in Vietnam, the majority (232 (70.9%)) were nurses.^9^ In Jordan, all the populations were dentists.^23^ The finding of Saudi Arabia revealed that nurses were about 200 (24.4%), physicians were 185 (22.6%), while the rest were other health care workers.^24^ Similarly, the study of Pakistan found that doctors were 29.98%, pharmacists were 46.55%, and nurse was 25.37%^18^ (Table 1).

**Table 1.** Summary of baseline characteristics of the articles that were previously published and included studies in the systematic review, 2020.

Primary author	Year of publication	Study design	Country (study setting)	Average age in years	Sample size	Gender (male %)	Occupation/educational level
**Zhong et al.** ^1^	2020	Cross-sectional	China	16–29 = 40.8% 3049 = 51.7% 50+ = 7.5%	6910	34.3%	Physical labor = 17.2% Unemployed = 6.5% Students = 20.1% Mental labor = 56.2%
**Wolf et al.** ^6^	2020	Cross-sectional survey linked to three active clinical trials	USA	62.1 ± 11.3	630	40.3%	Working for pay = 40.6% Not working (retired/unemployed = 59.4%
**Shi et al.** ^22^	2020	Cross-sectional	China	33.74 ± 8.08	311	64.95%	Psychiatrists = 45.34% Nurses = 54.66%
**Roy et al.** ^25^	2020	Cross-sectional	India	29.09 ± 8.83	662	48.6%	Graduates and above ⩾ 90% healthcare professionals =~50%
**Dost et al.** ^8^	2020	Cross-sectional	Turkey	21–30 = 25.1% 31–40 = 35.5% 41–50 = 30.9% 51–60 = 7.5% >6 = 0.9%	346	38.4%	Specialist = 50.6% Resident = 33.8% Professor = 5.5% Associate Professor = 4.0% Assistant Professor = 6.1%
**Geldsetzer** ^26^	2020	Cross-sectional	United States and United Kingdom	United States: 18–27 = 21.9%: 18.4% 28–37 = 23.0%: 18.6% 38–47 = 17.8%: 18.8% 48–57 = 16.5%: 16.1% ⩾58 = 20.8%: 28.0%	6000	United States: 49.1% United Kingdom: 48.8%	United States: Less than Bachelor’s degree = 46.4%: 48.5% Bachelor’s degree = 35.8%: 34.5% Master’s degree = 13.6%: 11.0% Doctorate = 2.2%: 2.7%
**Erfani et al.** ^27^	2020	Cross-sectional	Iran	34.37 ± 11.25 years	8591	33.6%	Health care related = 20.6% Non-health care related = 79.4%
**Hanafiah and Wan** ^28^	2020	Cross-sectional	Malaysia	<24 = 36.9% 25–44 = 44.3% ⩾45 = 18.8%	1075	37.1%	Primary and secondary% = 17.8% Diploma/undergraduate degree = 58.6% Postgraduate/professional degree = 23.6%
**Huynh et al.** ^9^	2020	Cross-sectional	Vietnam	30.1 ± 6.1	327	26.0%	Physician = 13.1% Nurse = 70.9% Pharmacist = 12.8% Technical staff = 3.1%
**Khader et al.** ^23^	2020	Cross-sectional	Jordan	32.9 ± 10.6	368	33.4%	Dentists = 100%
**Bener and Al-Khal** ^29^	2004	Cross-sectional	Qatar	<30 = 22.58% 30–39 = 44.66% 40–49 = 23.95% >50 = 8.80%	1386	51.95%	Illiterate = 2.38% Primary = 4.91% Secondary = 38.74% Preparatory = 10.97% University graduate = 43.0%
**Almutairi et al.** ^30^	2015	Cross-sectional	Saudi Arabia	18–24 = 40.9% 25–39 = 40.7% 40–59 = 16.4% ⩾60 = 1.9%	1147	61.9%	Non-educated = 2.4% Less than secondary = 14.3% Secondary = 29.5% University = 6.0% Higher = 7.8%
**Azlan et al.** ^17^	2020	Cross-sectional	Malaysia	34 ± 11.2	4850	58.9%	Student = 23.2% Private sector = 19.7% Self-employed = 5.5% Not employed = 4.0% Retiree = 2.0% Manual labor/contract = 0.7%
**Al-Hanawi et al.** ^14^	2020	Cross-sectional	Saudi Arabia	18–29 = 29.99% 30–39 = 27.74% 40–49 = 20.43% 50–59 = 13.93% ⩾60 = 7.91%	3388	41.97%	High school or below = 15.91% College/university degree = 56.20% Postgraduate degree = 27.89%
**Zhang et al.** ^19^	2020	Cross-sectional	China	–	1357	53.4%	Doctors = 36.5% Nurses = 46.5% Paramedics = 17%
**Lau et al.** ^31^	2020	Cross-sectional	Philippines	41.3 ± 14.6	2224	7.3%	No education = 2.2% Elementary = 41.8% high school = 46.4% college and above = 9.7%
**Reuben et al.** ^32^	2020	Cross-sectional	Nigeria	18–29 years = 44.3% 30–39 years = 36.3% 40–49 years = 15.8% 50–59 years = 3.6%	589	59.6%	High school = 4.4% College/bachelor = 59.1% Master = 26.0% PhD = 5.3%
**Saqlain et al.** ^18^	2020	Cross-sectional	Pakistan	<30 = 74.9% 31–39 = 16.7% 40–49 = 5.6% ⩾50 = 2.9%	414	50.5%	Doctor = 29.98% Pharmacist = 46.55% Nurse = 25.37%
**Maheshwari et al.** ^16^	2020	Cross-sectional	India	18–20 = 35.3% 21–23 = 54.5% ⩾24 = 10.2%	354	50.3%	Student = 100%
**Singh et al.** ^15^	2020	Cross-sectional	India	25.3 ± 4.1	231	34.6%	Student = 100%
**Asaad et al.** ^24^	2019	Cross-sectional	Saudi Arabia	33.7 ± 8.6	820	68.66%	Nurses = 24.4% Physicians = 22.6% Other health care workers = 53.0%

### Risk of bias and quality assessment

Randomization and allocation concealment was adequate in 14 articles and unclear in the remaining 7.^8,15,22,25,29,30,32^ Blinding of health care workers and general populations were unclear in 15 of the articles and adequate in the remaining 6,^9,14,24,29–31^ whereas blinding of outcomes assessment was adequate in 13 articles and unclear in the remaining 8 articles.^1,6,8,15,22,23,26,31^ Incomplete outcome data were obtained in 8 articles^8,9,15,22,23,25,26,31^ and the remaining were complete. In all articles involved, selective reporting and other bias were not obtained. Regarding to their quality assessment, 12 articles were good,^1,6,14,17–19,22,23,24,26,27,31^ 1 article was poor,^28^ and 8 were fair.^8,9,15,16,25,29,30,32^

### Patterns of COVID-19 KAP

#### Patterns of COVID-19 knowledge

Overall, the majority of the articles that were previously published had good knowledge about modes of transmission, clinical presentation, preventive strategy, incubation period, and use of quarantine. The study conducted in China revealed that about 90% and 89.51% of the articles that were previously published had good knowledge, respectively.^1,22^ In Nigeria, almost all of them had good knowledge.^32^ The finding of Iran and Qatar showed that about 96% and 79.4% had good knowledge about COVID-19 disease, respectively.^27,29^ However, the study conducted in Saudi Arabia and India revealed that only 51% and 40% of them had good knowledge, respectively.^15,24^ In the United States, about 71.7% of them knew the symptoms, and 69.8% of them knew prevention strategy.^6^ In Malaysia, about 68.5% of them knew as COVID-19 is a pandemic disease and about 93.5% of them knew as currently no vaccine to prevent the disease.^28^ Similarly, in Vietnam, about 67% of people knew about different modes of transmission, about 65.8% knew about the isolation period and 58.4% of them knew about COVID-19 treatment.^9^ On the contrary, one study conducted in Saudi Arabia showed that only half of them knew regarding the incubation period.^30^

#### Patterns of COVID-19 attitude

Regarding the attitude toward the COVID-19, a good attitude was reported. Accordingly, the study conducted in China showed that about 97.1% and 77.17% of them had a good attitude.^1,22^ Similarly, the study conducted in Saudi Arabia found that more than 70% of the health care workers had good attitude.^24^ However, the study conducted in Iran found a moderate attitude (60.8%) toward COVID-19.^27^ The studies conducted in the United States, Jordan, Qatar, China, and the Philippines found that they were worried about the disease itself, about spreading COVID-19 to others, and the economic impact of COVID-19.^6,19,28,29,31^ One study conducted in the United States, and United Kingdom participants found that they were fearful to eat food in the restaurants.^26^

#### Patterns of COVID-19 practice

In our review, we found the variable practice of the articles that were previously published toward combating COVID-19. The study done in China, Malaysia, Saudi Arabia, Nigeria, Pakistan, and India found good practicing skill toward COVID-19 prevention strategies.^1,14,16,18,19,28,30,32^ Other studies in Iran, United States, and United Kingdom, and Jordan found moderate practice.^23,26,27^ Finally, the previously published articles of the United States, Turkey, and Qatar had poor practice toward preventions of the disease.^6,8,29^

### Factors associated with COVID-19 KAP

In our review, different factors determined the KAP of COVID-19. The study done in Malaysia revealed that the associated factors include language, gender, age, education level, and employment status.^28^ The study conducted among Chinese residents showed that residents having high income and women had good KAP toward COVID-19.^1^

Another study conducted in Chinese psychiatric revealed that advanced training and work experience were determinants of COVID-19 KAP.^22^ On the contrary, a previously published articles of the United States showed that being black, poor, and had low health literacy were had a poor attitudes, and practice toward COVID-19.^6^

The study done in Iran showed that male gender, non-health care–related professions, single, and lower level of education were significantly associated with poor knowledge of COVID-19.^27^ Similarly, the study conducted in Vietnam, Pakistan, China, Saudi Arabia, and Malaysia revealed that occupation was a determinant of knowledge and attitude^9,14,17–19^ (Table 2).

**Table 2.** Summary of included studies on patterns of knowledge, attitude, and practice and associated factors of COVID-19.

Author	Knowledge	Attitude	Practice	Associated factors
**Zhong et al.** ^1^	Good = 90%	• Good = 97.1%	• Avoided crowded places = 96.4% • Wore masks = 98%	• Lower likelihood of negative attitudes and • preventive practices toward COVID-2019
**Wolf et al.** ^6^	• Know symptoms = 71.7% • Prevent infection = 69.8%	• “Very worried” about getting the coronavirus = 24.6%	• Ready to fight the outbreak = 20.8%	• Sex • Race • Economic status • Health literacy and • Day of survey
**Shi et al.** ^22^	• Extensive knowledge = 89.51%	• Good = 77.17%	–	• Advanced training and • Experience of caring for patients with COVID-19
**Roy et al.** ^25^	• Knows multiple modes of transmission = 29.5% • Regarded COVID-19 as a highly contagious disease = 43% • Acknowledged that washing hands frequently could stop the spread of infection = 97% • Regarded fever as a symptom of COVID-19 = 18.2%	• Agreed to quarantine = 96% • Thought social distancing is essential to stop the virus from spreading = 98% • Considered traveling within the country to be safe during the pandemic = 88.7%	–	–
**Dost et al.** ^8^	–	• Alcohol based hand antiseptics = 806% • Liquid soap = 67.3% • Sodium hypochlorite (1/10 diluted) = 37.0%, should be used as disinfectants.	• Started wearing masks after the pandemic because of fear of disease transmission = 43.9%	–
**Geldsetzer** ^26^	–	• Wearing a common surgical mask was “highly effective” = 37.8% of United States and 29.7% of United Kingdom • Prudent to refrain from eating at Chinese restaurants = 25.6% of United States and 29.6% of United Kingdom • Thought that children were at an especially high risk of death = 53.8% of United States and 39.1% of United Kingdom	• Recommended care-seeking option of staying home and contacting their • Good practice = 64.2% of United States and 79.0% of United Kingdom	–
**Erfani et al.** ^27^	• Good knowledge = 90%	• Had a moderate attitude = 60.8%	• Had moderate practice = 71.3%	• Male gender • Non-health care–related professions • Single and lower level of education
**Hanafiah and Wan** ^28^	• COVID-19 is a contagious respiratory disease = 98.9% agree • COVID-19 is caused by a bacteria • Called SARS-CoV-2 and can be treated with antibiotics = 64.7% disagree • COVID-19 is a zoonotic disease = 70.2% agree • COVID-19 is a pandemic disease = 68.5% agree • There is currently no approved vaccine to prevent COVID-19 = 93.5% agree	• Agreeing that COVID-19 is a very deadly disease = 79% • worried about themselves and loved ones getting sick with COVID-19 = 96.3% • Worried about spreading COVID-19 to others = 94.4% • Worried about the impact of COVID-19 on their work, livelihood and the economy = 96.1%	• Agreed they could reduce their risk of getting COVID-19 by avoiding crowded public areas, keeping their hands clean, and not touching their face = 99.1% • Agreed that closure of areas of congregations such as schools and places of worship are an extreme and unnecessary measure to control the spread of COVID-19 = 16.4%	• Language • Gender • Age • Education level and • Employment status.
**Huynh** et al.^9^	• Knew the mode of transmission = 67.0% • Knew the isolation period = 65.8% • Knew treatment = 58.4%	• Held positive attitude regarding the risk of personal = 82.3%, and family members = 79.8%	–	• Occupation
**Khader et al.** ^23^	Knew the incubation period is 1–14 days = 36.1%	• Perceived COVID-19 as very dangerous = 17.7% • Perceived it as moderately dangerous = 71.7% • Perceived it as not dangerous = 9.5%	• Had good practice on prevention of disease transmission = 74.7%	–
**Bener and Al-Khal** ^29^	Had good knowledge = 79.4%	• Afraid to travel for fear of being affected by SARS = 60%	• Poor level of practice = 31.7%	• Level of education
**Almutairi et al.** ^30^	• Not sure about their knowledge regarding incubation period = 50.5% • Not sure the period of communicability = 36.5% • Had good knowledge regarding quarantine = 86.2%	• Believed that there was a vaccine available for the disease = 25.5% • Aware that the disease was a viral illness = 91.6% • Mistakenly believed that the disease was an immunodeficiency disease 48.9%	• Hand washing = 94%, and the use of face masks in crowded areas = 74.9% • Avoiding touching their eyes, noses, or mouths = 81.3%	• Gender
**Azlan et al.** ^17^	• Good = 80.5%	• Positive attitudes = 83.1%	• Avoiding crowds = 83.4% • Proper hand hygiene = 87.8% • Wearing of face masks = 51.2%.	• Age • Religion • Occupation • Income • gender
**Al-Hanawi et al.** ^14^	• Good = 81.64%	• Positive attitudes = 94%	• Refrained from attending social events = 95% • Avoided crowded places = 94% • Avoided shaking hands = 88%	• Gender • Age • Marital status • Level of education • Occupation • Income level
**Zhang et al.** ^19^	• Good = 89%	• Worried = 85%	• Good = 89.7%	• Work experience • Occupation
**Lau et al.** ^31^	• Knows transmission route: • Coughing and sneezing = 89.5% • Indirect hand contact = 72.6% • Face to face talking = 83.0% • Handshakes or hugs = 81.2% • Sharing and eating from the same dish = 84.9%	• Worried = 80.3%	• Hand washing = 89.9% • Avoided crowded places = 62.9% • Social distancing = 32.4% • Wearing face masks = 28%	• Residence • Level of education • Having phone/television • Income
**Reuben et al.** ^32^	• Good knowledge = 99.5%	• Positive attitudes = 79.5%	• Practicing social distancing/self-isolation = 92.7% • Improved personal hygiene = 96.4% • Using face mask respectively 82.3%=	• Having good knowledge
**Saqlain et al.** ^18^	• Good knowledge = 93.2%	• Positive attitude (mean = 8.43)	• Good practice = 88.7%	• Occupation • Age • Year of experience
**Maheshwari et al.** ^16^	• Good knowledge = 92.7%	• Positive attitude ⩾ 80%	• Avoided unnecessary travel = 98.6% • Maintain social distance = 98.3% • Washing hands = 96.6% • used hand sanitizer = 92.7% • Wear face mask = 91.1%	• Gender
**Prasad Singh et al.** ^15^	• Good knowledge = 40%	• Good attitude about social distancing = 97.8% • Good attitude about lockdown strategy = 99.1%	• Regular hand washing/sanitizing using alcohol = 98.7% • Covering mouth and nose while coughing or sneezing = 97.4% • Social distancing = 97% • Staying at home = 97.8% • Cook meat and eggs well = 21.2% • Avoid unprotected direct contact with live animals = 30.3% • Seek hospital/health unit = 87%	–
**Asaad et al.** ^24^	• Had sufficient knowledge = 51%	• Exhibited a positive attitude ⩾ 70%	–	• Occupation

## Discussion

Good awareness of the modes of the transmission and preventive strategy of COVID-19 is a pivotal role to control the disease. Despite, this is determined by the people’s behavior toward COVID-19.^27^

In our study, the majority of the articles that were previously published had good knowledge of the COVID-19. However, about half and more than half of them had poor knowledge in Saudi Arabia and India.^24,15^ This is consistent with the study done in Addis Zemen Hospital, Northwest Ethiopia,^33^ Malaysia,^17^ Saudi Arabia,^14^ and healthcare workers in Henan, China.^19^

Regarding their attitude, the majority of them had a good attitude about COVID-19. However, the study conducted in Iran revealed that most of them (60.8%) had a moderate attitude.^27^ This is consistent with the finding of North-Central Nigeria,^32^ Pakistan,^18^ and India.^16^ The finding of the search in the United States, Malaysia, Jordan, Qatar, China, and the Philippines revealed that most of the articles that were previously published were worried about the disease itself.^6,19,23,28,29,31^

Another study also found that there is a reported increase in boredom, sadness, loneliness, and worry as the results of lockdown.^34^ An increased prevalence of depression (29.2%) was found predominately in patients who experienced COVID-19 infection.^35^ Health care providers were more likely to develop different psychological disorders like anxiety, depression, and posttraumatic stress disorder as the result of challenges and stress they experience during the management of COVID-19.^36^ Besides, the spread of the virus had resulted in the subsequent development of anxiety in the general population.^37^

Regarding their practice, wearing a face mask was widely practiced in the china, Turkey, and Saudi Arabia, Nigeria, and India to combat the disease,^1,8,16,30,32^ whereas good home staying and avoid crowded environment practice was common in the United States, United Kingdom, Malaysia, and India.^15,26,28^

This is inconsistent with the study of Jimma university medical center in which hand washing and avoidance of handshaking were a dominant practice.^38^ This different practice to avoid the disease was may be due to the difference in socio-demographic characteristics in the previously published articles.

This study found different determinants for KAP of COVID-19 which includes socio-demographic characteristics (age, gender economic status, race, marital status, occupation, and language).^6,9,14,17–19,24,27–30^ This is consistent with the study of Addis Zemen Hospital, Northwest Ethiopia.^33^

According to the study conducted in Vietnam, Pakistan, China, Saudi Arabia, and Malaysia, occupation was a determinant of knowledge and attitude.^9,14,17–19^ Similarly, occupation was a determinant of good knowledge and attitude in Jimma university medical center, southwest Ethiopia.^38^ On the contrary, the study done in Jordan indicated that there was no association between occupations.^39^

Generally, adopting good prevention and protection measures can possibly help overcome this COVID-19 pandemic.^40^ Therefore, every country should implement the strategy to combat the disease to increase the level of practice.

### Strength and limitations

As strength, the study was tried to assess the determinants of poor KAP among both general populations and health care providers. However, as a limitation, all included studies were cross-sectional, which was difficult to identify causal effect relationships. The other weakness includes the limited number of articles reached, quantitative analysis was not performed and heterogeneity of the articles.

## Conclusion

This systematic review found that the majority of the articles that were previously published had good knowledge about modes of transmission, clinical presentation, preventive strategy, incubation period, and use of quarantine. Despite the review of the studies showed good attitude toward COVID-19, the majority of the articles that were previously published were worried about the disease. Therefore, further research should be conducted to identify the psychological effect of COVID-19 on their mental health. Regarding their practice, we found variable practice in previously published articles toward combating COVID-19 (good, moderate, and poor practice). Several factors were associated with poor knowledge, poor attitudes, and poor practice skills in response to the epidemic of COVID-2019, such as level of education, occupation, income, gender, age, marital status, and race. Therefore, besides socio-demographic factors, other determinants of KAP should be studied at large.
